# Chikungunya virus infections in Finnish travellers 2009-2019

**DOI:** 10.1080/20008686.2020.1798096

**Published:** 2020-08-26

**Authors:** A. J. Jääskeläinen, L. Kareinen, T. Smura, H. Kallio-Kokko, O. Vapalahti

**Affiliations:** a HUS Diagnostic Center, HUSLAB, Clinical Microbiology, University of Helsinki and Helsinki University Hospital, Helsinki, Helsinki, Finland; b Department of Virology, Faculty of Medicine, University of Helsinki, Helsinki, Finland; c Department of Veterinary Biosciences, University of Helsinki, Helsinki, Finland

**Keywords:** Chikungunya, RT-qPCR, NGS, Thailand, diagnostics

## Abstract

The mosquito-borne chikungunya virus (CHIKV) causes an acute febrile illness with rash, joint and muscle pain.A realtime RT-PCR assay for CHIKV detecting non-structural protein (nsP2; CHIKV nsP2-RT-qPCR) was set up. All the serodiagnosed CHIKV cases detected during 2009-2019 in Finland were screened with the assay, followed by isolations attempts and sequencing using Sanger and next generation sequencing (NGS). To validate the assay external and in-house quality control samples were used and all were correctly identified. Specificity of the assay was 100%. Assay was sensitive to detect CHIKV RNA in dilution of 10^-8^.During years 2009-2019 34 patients were diagnosed for acute CHIKV infection. Twelve out of 34 cases were positive by CHIKV nsP2-RT-qPCR.Two CHIKV isolations succeeded from two individuals infected originally in Thailand, 2019. From 12 CHIKV nsP2-RT-qPCR positive samples, five (42%) CHIKVs were successfully sequenced. In this study, CHIKVs from year 2019 clustered with CHIKV ECSA-lineage forming sub-cluster with strains from ones detected in Bangladesh 2017, and the ones from Jamaica (2014) within Asian lineage showing highest similarity to strains detected in Caribbean outbreak 2013-15.  Majority of the CHIKV infections detected in Finland originates from Asia and virus lineages reflect the global circulation of the pathogen.

## Introduction

Diagnosis of febrile illnesses in travelers and people living in areas endemic for mosquito-borne diseases is a global challenge. There is a highly diverse group of etiological agents and diseases to consider, such as malaria, dengue, and enteric fevers [1]. Several of the mosquito-borne microbes are clinically important viruses, such as dengue (DENV), Zika (ZIKV), West Nile (WNV), and chikungunya viruses (CHIKV). Since tropically circulating diseases may cause life-threatening complications, the correct identification of the causative pathogen is of importance.

Globally one of the most significant mosquito-borne viruses is the CHIKV which belongs to the genus *Alphavirus* and causes millions of infections annually. CHIKV circulates in the tropical and subtropical regions of Africa, Asia, and the Americas and its main vector species are the *Aedes albopictus* and *A. aegypti* mosquitoes [2]. At the moment, three major genetic lineages are found: West African, East/Central/South African (ECSA) which includes the Indian Ocean lineage (IOL) that emerged in 2004, and the Asian lineage [3]. Large outbreaks have occurred in Asia, for example, in Thailand during the years 2008–2009, 2013, and 2018–2019, but also in the Americas during 2013–15, and more recently, in Brazil in 2017–18 [4–12]. An acute CHIKV infection has a mean incubation time of three days and the clinical symptoms include fever, rash, severe joint and muscle pain, and edema in the joints [13]. Infection is confirmed by either serology or molecular methods, but in endemic regions, the debilitating arthralgia can also be used for a fairly reliable clinical diagnosis [13].

In Finland, CHIKV infections occur only in travelers, reflecting the epidemiological situation in the endemic tropical areas. The continental subarctic climate of Finland is not suitable for the vector species of CHIKV [14], and consequently, there are no autochthonous cases of CHIKV in Finland, unlike the Mediterranean more precisely Italy and France [15–17].

HUSLAB (HUS Diagnostic center, Helsinki, Finland) performs the laboratory diagnostics of CHIKV infections in Finland. Thirty-four CHIKV infections have been serodiagnosed in Finnish travelers during the years 2009–2019. All of the collected samples were examined using CHIKV IgM ELISA (Euroimmun) and CHIKV nsp2-RT-qPCR followed by virus isolation, sequencing, and molecular epidemiology analysis. In addition, data from the validation of a real-time reverse-transcription PCR assay detecting CHIKV the non-structural protein (CHIKV nsP2-RT-qPCR) are provided.

## Materials and methods

We screened retrospectively all the samples sent to HUSLAB (Helsinki, Finland) and serodiagnosed for acute CHIKV infection using the CHIKV nsP2-RT-qPCR followed by Sanger sequencing and next-generation sequencing (NGS) for a whole-genome sequencing approach. Virus isolation was attempted from all CHIKV nsP2-RT-qPCR positive samples.

### Virus strains and samples for validation

In order to test the specificity of the CHIKV nsP2-RT-qPCR, a panel of 15 CHIKV antibody-negative human serum samples from Finnish individuals [originally tested negative using CHIKV hemagglutination inhibition tests and immunofluorescence assays, HUSLAB] was used. In addition, we tested RNA extracted from cell culture isolations of DENV 1–4 (Robert Koch Institute (RKI), Germany, and Zeptometrix Corporation, Buffalo, New York, USA), ZIKV (strain MR766, Zeptometrix), yellow fever virus (YFV, strains 17D and Asibi, RKI), and WNV (strain Eg101, in-house).

For validation and quality control purposes, we also tested CHIKV Caribbean (European Virus Archive) and Ross (in-house) strains as well as 12 external quality assessment (EQA) samples distributed by the European Network for Diagnostics of Imported Viral Diseases (EVD-Labnet). The EQA samples consisted of CHIKV strains H20235 Saint Martin/2013 (Asian genotype), 236 origin Seychelles and 3162 origin India (both of the East-Central-South-Africa genotype) as well as O’nyong-nyong virus (ONNV) strain Ahero, Sindbis virus (SINV) strain Edgar 339, and DENV-2 strain VR-345 (EQA). The sample set is described thoroughly by [18].

### Acute CHIKV disease samples

**Figure 1.**
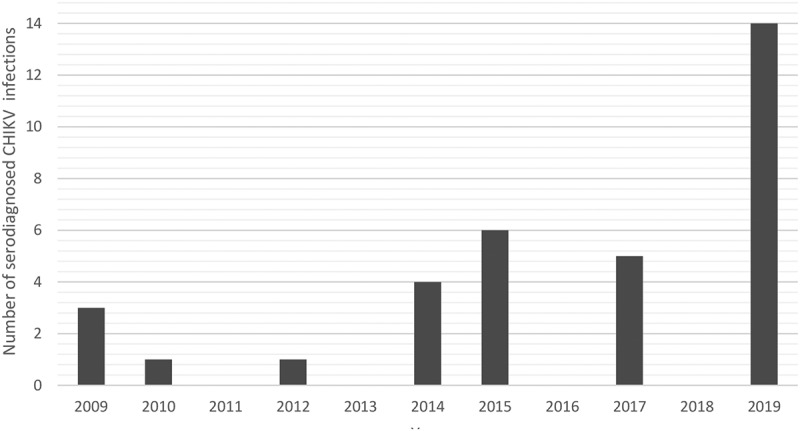
Annual variation of serodiagnosed acute CHIKV infections in Finland, years 2009–2019.

Altogether, 34 patients were diagnosed with acute CHIKV disease from 2009 until the end of December 2019 in Finland (Table 1). The annual variation of acute CHIKV cases in Finland is presented in Figure 1. The primary diagnosis has been made using serology, i.e. rise in IgG using IFA or hemagglutination inhibition titers (detects total antibodies) and positive IgM test, either using immunofluorescence and/or ELISA (Euroimmun) (Table 1). All of the patients were tested to be negative for acute dengue fever (HUSLAB). All patient samples were handled anonymously and according to research permits (of projects TYH2016258, TYH2017257, and TYH2019263) of Helsinki University Hospital.

**Table 1.** Demographic data and laboratory findings from patients with acute CHIKV disease during years 2009–2019.

Year	Sex (f/m)	Age (yEARS)	Travel history	Symptoms	Travel month; Sample taken days/weeks/months after onset of symptoms	Total Ab HI (titer)/IgG IFA:pos/neg (titer)	ELISA IgM (Euroimmun), pos ≥1.1	RT-qPCR neg/pos (Ct)	NGS/sequencing etc INFO
2009	f	28	Thailand (Asia)	Fever, rash, joint pain	June; >1 week	HI: 320	6.4	neg	ND
	f	41	*NA*	Joint pain, joint swelling	June; *NA*	HI: 320	2.6	neg	ND
	f	41	Singapore (Asia)	Fever, rash, mosquito bites	June; >1 week	HI: 160	ND	neg	ND
2010	m	27	Indonesia (Asia)	Fever [21]	November; <1 week	HI:2560	6.3	pos, Ct 34.01	Sequencing carried out by 21
*2011*	*No cases reported*								
2012	f	34	Papua New Guinea (Oceania)	Typical symptoms of CHIKV infection (not specified in more detail)	December, 2012; ~4 months	HI:80	2.9	ND	ND
*2013*	*No cases reported*								
2014	m	56	The Philippines (Asia)	*NA*	January; ~2 months	HI:320	3.2	ND	ND
	m	27	Grenada (Caribbean)	Fever, PNS symptoms, swollen lymph nodes	September; <1 week	HI:20	7.2	pos, Ct 41	RT-qPCR product was sequenced: CHIKV; sequencing using nested-RT-PCR/NGS or isolation not successful
	f	32	Jamaica (Caribbean)	*NA*	November; <1 week	HI:20	7.4	pos, Ct 31.7	Sequencing: CHIKV; Isolation not successful
					November; 15 days	HI:160	7.2	ND	ND
	m	33	Jamaica (Caribbean)	*NA*	November; <1 week	HI:<20	<1.1	pos, Ct 27.8	Sequencing/NGS: Whole CHIKV genome; Isolation attempts not succeeded.
					November; 17 days	HI:160	7.6	ND	ND
2015	f	33	Thailand (Asia)	Fever, rash, thrombocytopenia	January; >1 week	HI:160	1.1	neg	ND
	m	56	Indonesia and Bali, Singapore (Asia)	High fever (fluctuating), joint pain, rash	January; ~1 wk	HI:160	5.7	pos, Ct 34.9	RT-qPCR product was sequenced: CHIKV; sequencing using nested-RT-PCR/NGS or isolation not successful
	f	30	*NA*	*NA*	*NA; NA (sample taken in April)*	HI: 640	6.3	neg	ND
	f	29	Mexico (Caribbean coast)	Joint pain, tired	July; >1 week	HI: 1280	7.7	neg	ND
	f	48	Cuba (Caribbean)	Fever	November; >3 weeks	HI:320	6.5	neg	ND
	m	51	*NA*	*NA*	*NA; NA (sample taken in December)*	HI:640	6.3	neg	ND
*2016*	*No cases reported*								
2017/ 2018*	m	24	*NA*	*NA*	*NA; NA (sample taken in June)*	IFA:640	5.6	neg	ND
	m	25	Bangladesh (Asia)	Fever, itchy skin, joint pain, stomach ache	July; >2 weeks	IFA:1280	3.3	neg	ND
	m	31	Bangladesh (Asia)	Typical symptoms for CHIKV infection (not specified in more detail)	June and July; >3 weeks	IFA: >1280	7.2	neg	ND
	m	54	Bangladesh (Asia)	Fever, joint pain	June; >4 weeks	IFA:1280	4.0	neg	ND
	m	47	The Phillippines (Asia)	*NA*	December 2017/January 2018; >2 weeks	IFA:2560	4.9	neg	ND
*2018*									
2019	f	43	Thailand (Asia)	*NA*	January; NA	ND	ND	pos, Ct 24.54	Sequencing: CHIKV; Isolation not successful. NGS not performed
					January; >3 months (sample taken in April)	IFA: >2560	1.2	neg	ND
	f	35	Thailand (Asia)	*NA*	January; NA	IFA: >2560	6.4	neg	ND
	f	13	Thailand (Asia)	Fever, septic shock suspected, rash, arthralgia; treated in ICU in Thailand [34]	February; 11 days	IFA: >2560	7.1	pos, Ct 35.47	Serology and symptoms described in 34, sequencing and isolation not successful.
	f	49	Thailand [Asia)	Fever, joint pain, diarrhoea, rash	February; 10 days	IFA: >2560	6.9	pos, Ct 35.64	Serology and symptoms described in 34, sequencing and isolation not successful.
	m	9	Thailand [Asia]	Fever, rash, arthralgia, diarrhoea, underlying disease: type 1 diabetes	February; 11 days	IFA: >2560	7.3	pos, Ct 36.31	Serology and symptoms described in 34, sequencing and isolation not successful.
	m	70	Thailand [Asia]	Fever, arthralgia, polyarthritis, tenosynovitis, itching without rash, underlying disease: type 2 diabetes, hypertension, cardiovascular disease	February; >3 weeks	IFA: 1280	2.9	neg	Serology and symptoms described in 34
	m	47	Thailand (Asia]	Fever, arthralgia, strong pain in heels [inability to walk), itching without rash	February; >3 weeks	IFA: >2560	3.7	neg	Serology and symproms described in 34
	f	52	Thailand and Phuket (Asia]	Fever, joint pain	February; <1 week	IFA: <10	<1.1	pos, Ct 28.6	Sequencing/NGS: Whole CHIKV genome; CHIKV isolation.
					February; >2 months (sample taken in April)	IFA: >2560	3.2	ND	ND
	m	51	Thailand and Phuket (Asia)	Fever, rash, mild conjuctivitis, arthralgia, inability to walk, underlying disease: Legg-Calvé-Perthes disease	February; <1 week	IFA: <10	<1.1	pos, Ct 31.5	Sequencing/NGS: Whole CHIKV genome; CHIKV isolation
					February; >3 months (sample taken in May)	IFA:1280	4.9	ND	ND
	m	59	Thailand (Asia)	Fever, joint pain, light leucopenia and thrombocytopenia	March, <1 week	IFA: 160	5.1	pos, Ct 33.23	Sequencing and isolation not successful.
	f	24	Thailand (Asia)	NA	March, >3 months (sample taken in June)	IFA: >10 240	2.9	ND	ND
	m	31	*NA*	*NA*	NA; NA (sample taken in June)	IFA: >2560	7.3	neg	ND
	m	14	*NA*	*NA*	NA; NA (sample taken in July)	IFA: >2560	6.3	neg	ND
	f	51	Thailand (Asia)	NA	NA; NA (sample taken in July)	IFA: >2560	2.1	neg	ND

NA, not available; m, male; f, female; Total Ab HI, total antibody titer defined by hemagglutination inhibition test; IFA, immunofluorescence assay; pos, positive; neg, negative; Ct, cyclic threshold; NGS, next-generation sequencing; ND, not determined; ICU, intensive care unit; PNS, paraneoplastic neurologic syndromes.

### Extraction methods

Total nucleic acids were extracted from the serum and EDTA-blood samples using MagNA Pure LC instrument and Total Nucleic acid kit (Roche, Espoo, Finland). Total nucleic acid templates were used for nsP2-RT-qPCR and nested RT-PCR, both described below. For the processing of the nsP2-RT-qPCR reaction mixtures and total nucleic acid templates, QIAgility (Qiagen, Hilden, Germany) automated system was also used.

### CHIKV RT-PCRs

We set up an in-house CHIKV nsP2-RT-qPCR based on previously published PCR primers (Centers for Disease Control and Prevention (CDC)) [19] comprising a 101 bp long PCR product. Two detection probes were designed in this study, and different primers and probes are presented in more detail in Table 2.

**Table 2.** Primers and probes used in CHIKV RT-qPCR assay.

Primer/probe	Sequence (direction 5´→ 3´); gene area: nsP2 protein (product size 101 bp), location in CHIKV genome (GenBank ID: MH670649.1; NCBI), match (%)
CHIKV-F	GAG CAT ACG GTT ACG CAG ATA G^a^; 3859–3880, 100%
CHIKV-R1	TAC TGG TGA TAC ATG GTG GTT TC^a^; 3960–3938, 100%
CHIKV-R2	TGC TGG TGA CAC ATG GTG GTT TC^a^; 3960–3938, 87%
CHIKV-Probe1	FAM-AAT CTG CGT ACT GGG ACG T-MGBNFQ^b^; 3896–3914, 100%
CHIKV-Probe2	FAM-CTG CGT ATT GGG ACG CA -MGBNFQ^b^; 3899–3915, 78%

CHIKV, chikungunya virus; bp, base pair; F, forward; R, reverse; nsP2, nonstructural protein 2; ID, identification code; NCBI, National Center for Biotechnology Information, U.S. National Library of Medicine.

^a^Adapted from 19

^b^Designed in this study.

Real-time RT-PCR was performed using the SuperScript III Platinum One-Step qRT-PCR Kit (Invitrogen, Carlsbad, CA, USA) with 320 nM of the CHIKV-F primer, 240 nM of both CHIKV-R1 and CHIKV-R2 primers and 160 nM of each probe. Five µl of the extracted eluate was subjected to 25 µl PCR-reaction with cycling conditions of 1 cycle of 50°C for 30 min, 1 cycle of 95°C for 2 min, followed by 45 cycles of 95°C for 15 sec and 60°C for 50 sec.

For Sanger sequencing, nested RT-PCR primers described by [20] were used. Nested RT-PCR was carried out using a final concentration of 40 pmol of each primer and Superscript® III Platinum® One-step RT-PCR System (Invitrogen) with the final template amount of 5 µl. The positive PCR products were purified using AMPure XP beads (Beckman-Coulter, CA, USA) and sequenced using Sanger method (Helsinki University sequencing services, Helsinki, Finland).

### Virus isolation protocol

The CHIKV nsP2-RT-qPCR positive patient samples (serum/EDTA-blood) were inoculated to Vero cells in 2% FBS MEM with penicillin-streptomycin-nystatin. Seven days after the infection, RNA was extracted from the cultured cells using Viral RNA mini kit (Qiagen) and tested using CHIKV nsP2-RT-qPCR.

One sample (indicated in Table 1; from year 2010) was not used for the virus isolation attempt since the CHIKV detected in this sample (taken in 2010) has been previously described by [21].

### Next-generation sequencing, NGS

For NGS, the original serum samples were filtered through 0.4 µm columns (Millipore, Burlington, Massachusetts, US) and treated with a mixture of micrococcal nuclease (New England BioLabs Ipswich, Massachusetts, US) and benzonase (Millipore) for 1 hour at 37°C, followed by RNA extraction using Trizol reagent (Thermo Fisher Scientific, Massachusetts, USA). Ribosomal RNA was removed using a NEBNext rRNA Depletion Kit (New England BioLabs) according to the manufacturer’s protocol. The sequencing library was prepared using a NEBNext Ultra II RNA Library Prep Kit (New England BioLabs). For cell culture isolates, RNA was extracted using Trizol reagent, followed by sequencing library preparation as described above. The library fragment sizes were measured using agarose gel electrophoresis and the concentrations using Qubit dsDNA BR Assay Kit (Life Technologies, Carlsbad, California, US) and NEBNext Library Quant Kit for Illumina (New England BioLabs). Sequencing was conducted using MiSeq Reagent Kit V2 with 150 bp reads.

Raw sequence reads were trimmed and low-quality, quality score <30, and short, <50 nt, sequences were removed using Trimmomatic [22]. Thereafter, de novo assembly was conducted using MegaHit [23]. Contigs were annotated using Blastn followed by re-assembly against the de-novo assembled CHIKV consensus sequences using BWA-MEM algorithm [24] implemented in SAMTools version 1.8 [25].

**Figure 2.**
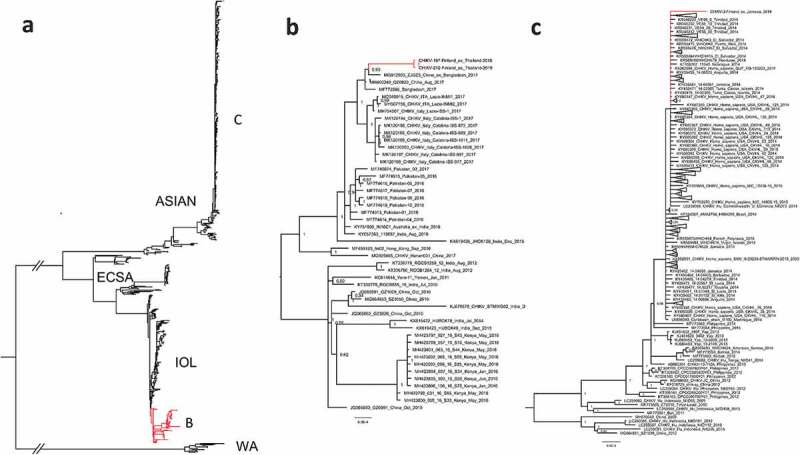
A phylogenetic tree of complete CHIKV coding sequences detected during 2009–2019. (a) All complete CHIKV coding sequences acquired from GenBank. 2(b) CHIKV strains from Thailand clustered within ECSA-lineage (c) CHIKV strain from Jamaica clustered within the Asian lineage.

For phylogenetic analysis, all complete CHIKV coding sequences were downloaded from the GenBank (National Center for Biotechnology Information, NCBI). Identical sequences were removed using CD-HIT [26] followed by alignment using ClustalW algorithm [27] implemented in MEGA7 [28]. The phylogenetic tree (Figure 2(a-c)) was constructed using the Bayesian Markov chain Monte Carlo (MCMC) method, implemented in Mr Bayes version 3.2 [29] using a GTR-G-I model of substitution with 2 independent runs and 4 chains per run. The analysis was run for 5 million states and sampled every 5000 steps.

A phylogenetic tree was constructed using the Bayesian Markov chain Monte Carlo (MCMC) method, implemented in Mr Bayes version 3.2 using a GTR-G-I model of substitution with 2 independent runs and 4 chains per run.

## Results

### CHIKV nsP2-RT-qPCR

The negative serum panel (n = 15) and controls representing other viruses (DENVs, ZIKV, YFVs, WNV, ONNV and SINV) all tested as negative by CHIKV nsP2-RT-qPCR, showing 100% specificity. In order to evaluate the analytical sensitivity of the assay, external EQA (EVD-Labnet) samples and CHIKV RNA dilutions were tested (Table 3). The CHIKV nsP2-RT-qPCR correctly identified all CHIKV positive EQA samples (n = 12; 100%), as well as the different ten-fold dilutions (10^−5^-10^−8^) of RNA extracted from cell culture supernatants of CHIKV strains Caribbean and Ross (100%). The median Ct values (five parallel reactions; standard deviation, SD; coefficient variation, CV%) for CHIKV Caribbean and Ross dilutions are presented in Table 3. The limit of detection was not calculated as there were no commercial or in-house purified and quantified CHIKV isolates or RNA available. However, the Ct value of 37 was considered the cut-off for positivity based on the parallel tests (Table 3). If the Ct value was over 37, the test was repeated and positivity was confirmed with other methods.

**Table 3.** Cycle threshold values of two different CHIKV dilution series using the nsP2 CHIKV RT-qPCR.

Virus	Dilutions tested, 10-fold	No of posit (x/5 parallel tests)	Median Ct value	SD	CV%
CHIKV Caribbean	10^−5^	5/5	25.29	0.19	0.5%
	10^−6^	5/5	29.95	0.08	0.3%
	10^−7^	5/5	33.53	0.27	0.8%
	10^−8^	1/5	35.14	ND	ND
CHIKV Ross	10^−5^	5/5	26.74	0.12	0.5%
	10^−6^	5/5	28.65	0.22	0.8%
	10^−7^	5/5	31.62	0.20	0.6%
	10^−8^	5/5	36.50	0.35	1.0%

CHIKV, chikungunya virus; posit, positives; ND, not determined; Ct, cycle threshold value; SD, standard deviation; CV, coefficient variation.

### Clinical samples

Demographic data and laboratory findings are presented in Table 1. The most frequent source of infection for Finnish patients was Asia, accounting for ~60% percent of cases, and particularly Thailand (Table 1). This most likely simply reflects the amount of Finnish travelers to Asia compared to other CHIKV endemic areas. The median age of the patients was 35 years (range: 9–70 years) and 16 of the patients were female and 18 were male.

Twelve of the acute CHIKV disease patients (n = 34, Table 1, Figure 1), were positive using the CHIKV nsP2-RT-qPCR. From these CHIKV nsP2-RT-qPCR positive cases, five (42%) were successfully downstream processed using Sanger sequencing and/or NGS. Altogether three whole genomes were constructed using NGS (Figure 2(a-c)). In this study, the whole-genome sequences obtained originated in the Caribbean (Jamaica 2014; 33-year-old male) and from Thailand (Phuket 2019; 52-year-old female and 51-year-old male). In addition, from two samples, CHIKV was sequenced using nested-RT-PCR (Sanger sequencing).

In the phylogenetic analysis, the strains from Thailand clustered within ECSA-lineage and formed a sub-cluster together with strains from Bangladesh and a Chinese traveler from Bangladesh 2017 Figure 2(b); [30, 31]. These strains formed a sister clade for CHIKV strains sequenced during a CHIKV outbreak in Italy in 2017 [32].

The strain from Jamaica clustered within the Asian lineage and showed the highest similarity with strains sequenced from a Caribbean outbreak during 2013–2015 Figure 2(c); [33].

The female patient from Thailand had reported fever and joint pain as clinical symptoms and the male patient had reported fever, rash, mild conjunctivitis, arthralgia, and the inability to walk. The male patient also had Legg-Calvé-Perthes disease as a pre-existing condition. No clinical symptoms or other information was available for the patients who contracted the infection in Jamaica.

We isolated CHIKVs from two CHIKV-seronegative patient samples (see serological data from Table 1) in Vero cells and confirmed the isolation by CHIKV nsP2-RT-qPCR and NGS from the cell culture supernatant. Both of the isolates originated in Phuket, Thailand, in 2019 (CHIKV-197-Finland_ex_Thailand-2019, MN075149; CHIKV-212-Finland_ex_Thailand-2019, MN075150). The sequences were identical (Figure 2(a)).

None of the other virus isolation attempts out of altogether 11 attempts were successful. Virus isolation did not succeed from an EDTA blood sample (2019; country visited: Thailand) which was positive for CHIKV RNA (Ct 24.5). As there is no validated protocol for NGS, an NGS run was not carried out in this sample.

## Discussion

The CHIKV nsP2-RT-qPCR reaction and protocol were optimized for automated sample preparation and PCR pipetting robots [MagNA Pure LC instrument (Roche) and QIAgility, Qiagen], and was validated in diagnostic laboratory settings (HUSLAB). The validated CHIKV nsP2-RT-qPCR showed excellent performance with high specificity (100%) and good sensitivity and specificity with the EQA samples tested. The study was limited by not having a calculated limit of detection value due to the fact that commercial and quantified CHIKV RNA was not available at the time of the study. However, the CHIKV RNA was detected in a dilution of 10^−8^ and nsP2-RT-qPCR excellent intra-assay repeatability (Table 3). Overall, the data show that this CHIKV nsP2-RT-qPCR can be used as a diagnostic assay for the detection of acute CHIKV disease in the early phase of the infection.

Thirty-four travel-associated CHIKV cases have been diagnosed in Finland during 2009–19 (Table 1). 2019 had the highest number of CHIKV cases (Figure 1 and Table 1). In total, 14 (41.2%; 14/34) acute cases were diagnosed during that year. We collected all serum/blood samples available from all of the 34 patients and tested them with the CHIKV nsP2-RT-qPCR, and subsequently attempted isolation and Sanger/NGS sequencing from CHIKV nsP2-RT-qPCR-positive samples. Twelve CHIKV nsP2-RT-qPCR positive samples were detected, and out of these 12, two virus isolations succeeded and three whole-genome sequences were obtained (Figure 2(a-c)).

In Finnish travelers, most of the CHIKV samples positive with CHIKV nsP2-RT-qPCR were taken less than one week after the onset of symptoms. Overall, the symptoms were typical for chikungunya and no fatal CHIKV disease cases have been reported in Finland (Table 1).

Nine Finnish patients returning from Thailand with severe symptoms have been described previously [34]. In these cases, at 10–11 days after the onset of symptoms, a low level of CHIKV RNAemia was still detected in three individuals using CHIKV nsP2-Rt-qPCR.

In 2019, all the CHIKV strains detected from Finnish travelers originated in Thailand and clustered within ECSA-lineage, subclustering with strains identified in 2017 in Bangladesh [30,31]. In addition, these strains formed a sister clade with the ones reported by [32] in Italy, 2017.

In 2014–2015, there was a peak of acute CHIKV cases (Figure 1) acquired from Asia and the Caribbean. During this time large CHIKV outbreaks were reported in the Americas as the CHIKV Asian lineage was introduced to and spread widely across the Caribbean, South and Central America, and the USA (Florida) [33]. These epidemics were also reflected in Finnish travelers. The detected CHIKV strains from years 2014–15 belonged to Asian lineage and had the highest similarity with the strains reported to cause the outbreak in the Caribbean during 2013–15.

When using serological methods in the diagnosis of acute CHIKV infection, samples from acute and convalescent phase are needed. This can postpone the diagnosis of CHIKV infection. The molecular-based diagnostics, however, can be used particularly in the early phase and the diagnosis can be confirmed earlier than using serological methods. In addition, samples with CHIKV genome detected can be further used for sequencing and surveillance of CHIKV strains circulating. This is as important as vector-surveillance for monitoring CHIKV spreading to new areas or new strains evolving.

## Author statements

The authors have declared no special author statements.

## Key results

- CHIKV strains were isolated and whole genomes were sequenced.
- A realtime RT-qPCR (nsP2-RT-qPCR) was set up and validated with Finnish sample panels with excellent specificity and sensitivity.
- Automated nucleic acid extraction followed by automated pipetting system was tested for CHIKV diagnostics.

## References

[1] Scaggs Huang FA , Schlaudecker E. Fever in the returning traveler. Infect Dis Clin North Am. 2018;32(1):163–9. 29406974 10.1016/j.idc.2017.10.009 PMC7135112

[2] World Health Organization (WHO) . [cited 2020 Jul 6 ]. https://www.who.int/news-room/fact-sheets/detail/chikungunya

[3] Caglioti C , Lalle E , Castilletti C , et al. Chikungunya virus infection: an overview. New Microbiol. 2013;36(3):211–227. 23912863

[4] Aubry M , Teissier A , Roche C , et al. Chikungunya outbreak, French Polynesia, 2014. Emerg Infect Dis. 2015;21(4):724–726. 25811534 10.3201/eid2104.141741 PMC4378499

[5] Beau F , Lastère S , Mallet H-P , et al. Impact on blood safety of the last arboviruses outbreaks in French Polynesia (2012–2018). Transfus Clin Biol. 2020;27(1):4–9. 31889619 10.1016/j.tracli.2019.12.001

[6] Cao-Lormeau VM , Musso D . Emerging arboviruses in the Pacific. Lancet. 2014;384(9954):1571–1572. 25443481 10.1016/S0140-6736(14)61977-2

[7] Chansaenroj J , Wanlapakorn N , Ngamsaithong C , et al. Genome sequences of chikungunya virus isolates from an outbreak in southwest Bangkok in 2018. Arch Virol. 2020;165(2):445–450. 31834526 10.1007/s00705-019-04509-1

[8] Lanciotti RS , Valadere AM . Transcontinental movement of Asian genotype chikungunya virus. Emerg Infect Dis. 2014;20(8):1400–1402. 25076384 10.3201/eid2008.140268 PMC4111183

[9] Leparc-Goffart I, Nougairede A, Cassadou S, et al. Chikungunya in the Americas. Lancet. 2014;383:514. 24506907 10.1016/S0140-6736(14)60185-9

[10] Pereira Gusmão Maia Z , Mota Pereira F , Do Carmo Said RF , et al. Return of the founder Chikungunya virus to its place of introduction into Brazil is revealed by genomic characterization of exanthematic disease cases. Emerg Microbes Infect. 2020;9(1):53–57. 31880218 10.1080/22221751.2019.1701954 PMC6968431

[11] Pongsiri P, V Auksornkitti, A Theamboonlers A, et al. Entire genome characterization of chikungunya virus from the 2008–2009 outbreaks in Thailand. Asian Pac J Tropical Biomedicine. 2010;27:167–176. 20962712

[12] Weaver SC , Forrester NL . Chikungunya: evolutionary history and recent epidemic spread. Antiviral Res. 2015;120:32–39. 25979669 10.1016/j.antiviral.2015.04.016

[13] Weaver SC , Lecuit M , Campion EW . Chikungunya Virus and the Global Spread of a Mosquito-Borne Disease. N Engl J Med. 2015;372(13):1231–1239. 25806915 10.1056/NEJMra1406035

[14] European Centre for Disease Prevention and Control and European Food Safety Authority . Mosquito maps [internet]. Stockholm: ECDC; 2020. [cited Jun 29 ]. Available from https://ecdc.europa.eu/en/disease-vectors/surveillance-and-disease-data/mosquito-maps

[15] Grandadam M , Caro V , Plumet S , et al. Chikungunya virus, southeastern France. Emerg Infect Dis. 2011;17(5):910–913. 21529410 10.3201/eid1705.101873 PMC3321794

[16] Rezza G , Nicoletti L , Angelini R , et al. Infection with chikungunya virus in Italy: an outbreak in a temperate region. Lancet. 2007;370(9602):1840–1846. . 18061059 10.1016/S0140-6736(07)61779-6

[17] Riccardo F , Venturi G , Di Luca M , et al. Secondary autochthonous outbreak of Chikungunya, Southern Italy, 2017. Emerg Infect Dis. 2019;25(11):2093–2095. 31625839 10.3201/eid2511.180949 PMC6810187

[18] Jacobsen S , Patel P , Schmidt-Chanasit J , et al. External quality assessment studies for laboratory performance of molecular and serological diagnosis of Chikungunya virus infection. J Clin Virol. 2016;76:55–65. 26828561 10.1016/j.jcv.2016.01.008

[19] Johnson BW , Russell BJ , Goodman CH . Laboratory diagnosis of Chikungunya virus infections and commercial sources for diagnostic assays. J Infect Dis. 2016;214(suppl 5):S471–S474. 27920176 10.1093/infdis/jiw274 PMC5657184

[20] Sánchez-Seco MP , Rosario D , Quiroz E , et al. A generic nested-RT-PCR followed by sequencing for detection and identification of members of the alphavirus genus. J Virol Methods. 2001;95(1–2):153–161. 11377722 10.1016/s0166-0934(01)00306-8

[21] Kurkela S , Sane J , Deren E , et al. Chikungunya virus as a causative agent of fever of unknown origin in Finnish travellers to tropics. J Clin Virol. 2012;54(3):289–290. 22459001 10.1016/j.jcv.2012.02.027

[22] Bolger AM , Lohse M , Usadel B . Trimmomatic: a flexible trimmer for Illumina sequence data. Bioinformatics. 2014;30(15):2114–2120. 24695404 10.1093/bioinformatics/btu170 PMC4103590

[23] Li D , Liu C-M , Luo R , et al. MEGAHIT: an ultra-fast single-node solution for large and complex metagenomics assembly via succinct de Bruijn graph. Bioinformatics. 2015;31(10):1674–1676. 25609793 10.1093/bioinformatics/btv033

[24] Li H . Aligning sequence reads, clone sequences and assembly contigs with BWA-MEM. Cite as arXiv:1303.3997. 2013. https://www.researchgate.net/publication/236935760_Aligning_sequence_reads_clone_sequences_and_assembly_contigs_with_BWA-MEM/citation/download.

[25] Li H , Handsaker B , Wysoker A , et al. The sequence alignment/map format and SAMtools. Bioinformatics. 2009;25(16):2078–2079. 19505943 10.1093/bioinformatics/btp352 PMC2723002

[26] Li W , Godzik A . Cd-hit: a fast program for clustering and comparing large sets of protein or nucleotide sequences. Bioinformatics. 2006;22(13):1658–1659. 16731699 10.1093/bioinformatics/btl158

[27] Thompson JD , Higgins DG , Gibson TJ . CLUSTAL W: improving the sensitivity of progressive multiple sequence alignment through sequence weighting, position-specific gap penalties and weight matrix choice. Nucleic Acids Res. 1994;22(22):4673–4680. 7984417 10.1093/nar/22.22.4673 PMC308517

[28] Kumar S , Stecher G , Tamura K . MEGA7: molecular evolutionary genetics analysis version 7.0 for bigger datasets. Mol Biol Evol. 2016;33(7):1870–1874. 27004904 10.1093/molbev/msw054 PMC8210823

[29] Rönquist F , Teslenko M , van der Mark P , et al. MrBayes 3.2: efficient Bayesian phylogenetic inference and model choice across a large model space. Syst Biol. 2012;61(3):539–4227. 22357727 10.1093/sysbio/sys029 PMC3329765

[30] Pan J , Fang C , Yan J , et al. Chikungunya fever outbreak, Zhejiang Province, China, 2017. Emerg Infect Dis. 2019;25(8):1589–1591. 31310205 10.3201/eid2508.181212 PMC6649353

[31] Pyke AT , Moore PR , McMahon J . New insights into chikungunya virus emergence and spread from Southeast Asia. Emerg Microbes Infect. 2018;7(1):1–3. 29535302 10.1038/s41426-018-0024-2 PMC5849737

[32] Lindh E, Argentini C, Remoli M.E., et al. The Italian 2017 Outbreak Chikungunya virus belongs to an emerging aedes albopictus-adapted virus cluster introduced from the Indian Subcontinent. Open Forum Infect Dis. 2018;12:6:ofy321. 10.1093/ofid/ofy321 PMC6345083 30697571

[33] Tan Y , Pickett BE , Shrivastava S , et al. Differing epidemiological dynamics of Chikungunya virus in the Americas during the 2014-2015 epidemic. PLoS Negl Trop Dis. 2018;12(7):e0006670. 30059496 10.1371/journal.pntd.0006670 PMC6085065

[34] Kantele A . Travellers as sentinels of chikungunya epidemics: a family cluster among Finnish travellers to Koh Lanta, Thailand, January 2019. Eurosurveillance. 2019;24(11). DOI:10.2807/1560-7917.ES.2019.24.11.1900162 PMC6425551 30892179

